# LT-YO-AN: lightweight YOLOv12 with asymmetric neck for apple fruitlet detection in complex orchard environments

**DOI:** 10.3389/fpls.2026.1958019

**Published:** 2026-10-14

**Authors:** Dandan Wang, Bo Wang

**Affiliations:** 1 College of Communication and Information Engineering, Xi’an University of Science and Technology, Xi’an, China; 2 Xi’an Key Laboratory of Network Convergence Communication, Xi’an, China; 3 School of Automation Science and Engineering, Faculty of Electronic and Information Engineering, Xi’an Jiaotong University, Xi’an, China

**Keywords:** asymmetric network architecture, high-resolution feature enhancement, Model lightweighting, pre-thinning apple detection, YOLOv12n

## Abstract

Accurate detection of apple fruitlets in complex orchard environments is a prerequisite for precision fruit thinning but remains challenging due to small target sizes, variable illumination, and dense foliage. To address these issues, a lightweight YOLOv12 architecture, termed LT-YO-AN, was proposed. The backbone integrates RepDown re-parameterizable downsampling and D3C2f aggregation modules, enabling hierarchical feature extraction with reduced spatial redundancy and channel-level compression. An asymmetric neck is introduced to explicitly enhance the high-resolution P2 layer for small-target sensitivity, while deliberately weakening the P5 layer via channel reduction (256 to 128) to alleviate redundancy. This design is complemented by a four-scale detection head (P2–P5) and a width scaling strategy (factor = 0.25), which together allocate computational resources preferentially to shallow features, yielding a compact model optimized for apple fruitlet detection. Evaluations on a real-world orchard dataset demonstrate that LT-YO-AN achieves an mAP50–95 of 0.675 and a recall of 0.860, outperforming benchmark YOLO variants. Although the computational complexity increases to 11.5 GFLOPs, the model maintains a compact size of 3.5 MB with 1.49 M parameters, making it suitable for edge deployment. This study provides a viable technological solution for early-stage fruit detection and automated orchard management.

## 1 Introduction

Precision fruit thinning is a critical agronomic practice in modern apple (Malus domestica) orchards, directly determining final fruit quality, yield distribution, and return bloom in subsequent seasons (Robinson and Lakso, 2008; Greene et al., 2013). Unlike mature fruit harvesting, pre-thinning operations must be performed when apples are still in their small-fruitlet stage, typically measuring 10~25 mm in diameter (Rufato et al., 2014; Li et al., 2026). At this stage, fruits exhibit chromatic similarity to surrounding foliage and are frequently obscured by branches and dense foliage, presenting extreme challenges for vision-based detection systems deployed on autonomous orchard robots (Silwal et al., 2016; Qureshi et al., 2023). Accurate, real-time localization of these fruitlets is therefore a prerequisite for thinning actuators; nevertheless, achieving reliable detection under such severe appearance-based interference remains a critical impediment to the widespread adoption of automated horticultural systems (Mirbod et al., 2023).

Deep learning-based object detection has progressively replaced traditional color-segmentation and handcrafted-feature methods in agricultural vision tasks (Koirala et al., 2019; Gené-Mola et al., 2020; Sun, 2024; Wang et al., 2026; Lei et al., 2026; Yu et al., 2026). Two-stage detectors such as Faster R-CNN (Ren et al., 2016) and Mask R-CNN (He et al., 2017) achieve high accuracy but suffer from excessive computational overhead, making them ill-suited for edge-deployed orchard platforms (Wan and Goudos, 2020; Wang and He, 2022). Single-shot detectors, particularly the YOLO (You Only Look Once) series, have become the dominant approach for real-time fruit detection due to their favorable speed-accuracy trade-off (Redmon et al., 2016; Bochkovskiy et al., 2020). Recent iterations, YOLOv8 through YOLOv12, have refined CNN-based backbones, introduced improved feature pyramids, and incorporated area-attention mechanisms to boost performance in complex scenes (Tian et al., 2026; Sapkota and Karkee, 2025). YOLOv12, in particular, adopts an attention-centric architecture built upon residual efficient layer aggregation networks (R-ELAN), achieving state-of-the-art mAP on COCO while maintaining sub-10 ms latency on embedded GPUs (Tian et al., 2026).

Despite these advances, deploying standard YOLO variants for pre-thinning apple detection faces three interrelated constraints. First, the intrinsic small-target nature of fruitlets leads to severe feature dilution across successive strided convolutions; by the P5 (stride=32) layer, the spatial footprint of these fruitlets diminishes to sub-pixel scales, effectively dissolving into background noise and severely weakening activation signals, even though shallower layers may retain partial cues (Kim et al., 2025). Second, agricultural edge devices, such as Jetson Nano, Raspberry Pi 5, or orchard UGV onboard controllers, impose strict limits on parameters, FLOPs, and memory bandwidth, necessitating ultra-lightweight architectures without catastrophic precision drop (Ye et al., 2025; Ma et al., 2025). Third, conventional YOLO necks employ symmetric feature pyramids (P3–P5 or P2–P5) that allocate uniform computational resources across scales. During this specific phenological phase, the visual field is dominated by high-density populations of small fruitlets, rendering uniform resource allocation inefficient (Long et al., 2023; Liu et al., 2018; Rufato et al., 2014).

To address lightweighting, recent studies have explored pruning, knowledge distillation, depthwise separable convolutions, and reparameterization strategies within YOLO frameworks for fruit detection (Tan and Le, 2021; Luan et al., 2026). For instance, YOLO-SPM replaces A2C2f with partial convolutions and integrates SimAM attention to reduce parameters while improving apple mAP in dwarf rootstock orchards (Li et al., 2026). AHG-YOLO applies ADown downsampling and group-convolution heads for lightweight pear detection, achieving 94.1% mAP50 at 5.1 MB (Ma et al., 2025). Similarly, S-YOLO leverages GSConv_SlimNeck and SE attention for greenhouse tomato detection with less than 9.2 M parameters (Sun, 2024). However, most agricultural lightweight YOLOs merely transplant generic compression strategies without rethinking multi-scale allocation asymmetry. Addressing this superficially, some introduce an additional P2 head to improve small-target sensitivity; however, this additive approach merely expands the network without commensurate reductions elsewhere, resulting in significant computational overhead. Others rely on heuristic pruning that degrades deep semantics essential for occlusion reasoning unless compensated by distillation (Wang and He, 2021).

A parallel line of advancement is the emergence of structurally optimized ultra-lightweight YOLOs beyond manual pruning. The recent YOLO-ULM proposes two targeted modules, D3C2f and RepDown, to reduce parameters while preserving high-level semantics and edge texture (Han et al., 2026). YOLO-ULM demonstrates that optimizing where and how downsampling and aggregation occur yields better efficiency than blind channel reduction. Nevertheless, YOLO-ULM is designed for generic COCO-scale objects. Its default head configuration and balanced pyramid overlook the extreme small-target bias of pre-thinning apples, where coarse stride-32 semantics contribute marginally to fruitlet localization (Kim et al., 2025).

Prior works on small apple fruitlet detection partially address this gap. Studies on YOLO11n-CBAM for young Redlove apples incorporate dual-dimensional attention to improve occlusion robustness but retain standard three-head detection with moderate parameters (Fan et al., 2026). Evaluations across YOLOv8~YOLOv12 for fruitlet counting in Washington State orchards show YOLOv9 and YOLO11n excel in accuracy and speed respectively, yet all baselines use symmetric pyramids unsuited for nano-scale deployment (Sapkota et al., 2026). UAV-based YOLO-LT adds dilated residual segmentation and focal modulation for orchard yield prediction but operates at higher resolution and compute budgets unsuitable for ground robots (Dong and Liu, 2025). Collectively, these efforts reveal a persistent trilemma: light-weighting via pruning sacrifices small-target sensitivity; enhancing P2 indiscriminately inflates latency; retaining symmetric P5 wastes resources on irrelevant large-scale features.

To break this trilemma, we posit that computational asymmetry is paramount for pre-thinning detection. Moving beyond merely adopting efficient modules, we redesign the feature pyramid topology to establish an asymmetric multi-scale feature enhancement strategy. By integrating YOLO-ULM’s components as foundational building blocks, we construct a tailored YOLOv12n variant that reallocates computational budgets: reinforcing high-resolution shallow pathways (P2) to recover vanishing fruitlet cues, while systematically attenuating deep redundant pathways (P5) via channel compression. Our contributions are as follows:

1. Identification of computational asymmetry in pre-thinning fruitlet detection and the proposal of an asymmetric multi-scale feature enhancement strategy.
2. Selective reinforcement of the P2 pathway via enhanced upsampling and D3C2f stacking, thereby recovering spatially vanishing fruitlet cues without incurring prohibitive overhead.
3. Systematic attenuation of the P5 pathway through channel-compressed RepDown, eliminating redundant computation.
4. Rigorous validation on a real-world orchard dataset, achieving not only superior small-target mAP against state-of-the-art baselines but also high deployability on edge devices for autonomous orchard robots.

The remainder of this paper is organized as follows: Section 2 describes the acquisition of the field dataset and the proposed LT-YO-AN architecture in detail. Section 3 presents the experimental setup and evaluation metrics. Section 4 presents a comprehensive performance evaluation, encompassing comparative benchmarks against state-of-the-art baselines and systematic ablation studies. Section 5 discusses the implications of these findings and analyzes representative failure cases encountered in complex orchard environments. Finally, Section 6 concludes the study and suggests directions for future research.

## 2 Materials and methods

### 2.1 Dataset preparation

The dataset utilized in this study was acquired from the experimental orchard at the College of Horticulture, Northwest A&F University, Yangling, Shaanxi, China. Images were captured using an iPhone 7 Plus under real-world orchard conditions to ensure ecological validity. Data collection was conducted over three sessions in early May 2018, specifically May 1 (sunny), May 2 (sunny), and May 4 (cloudy), with daily acquisitions scheduled between 09:00–11:30 and 14:30–18:30. This protocol facilitated the capture of diverse illumination conditions, including backlight and direct sunlight scenarios. At the time of acquisition, apple fruitlets were in their early developmental stage, characterized by horizontal diameters of less than 25 mm. All images were recorded in JPEG format at a resolution of 3024 × 3024 pixels. Example images captured in the orchard are shown in **Figure 1**.

**Figure 1:**
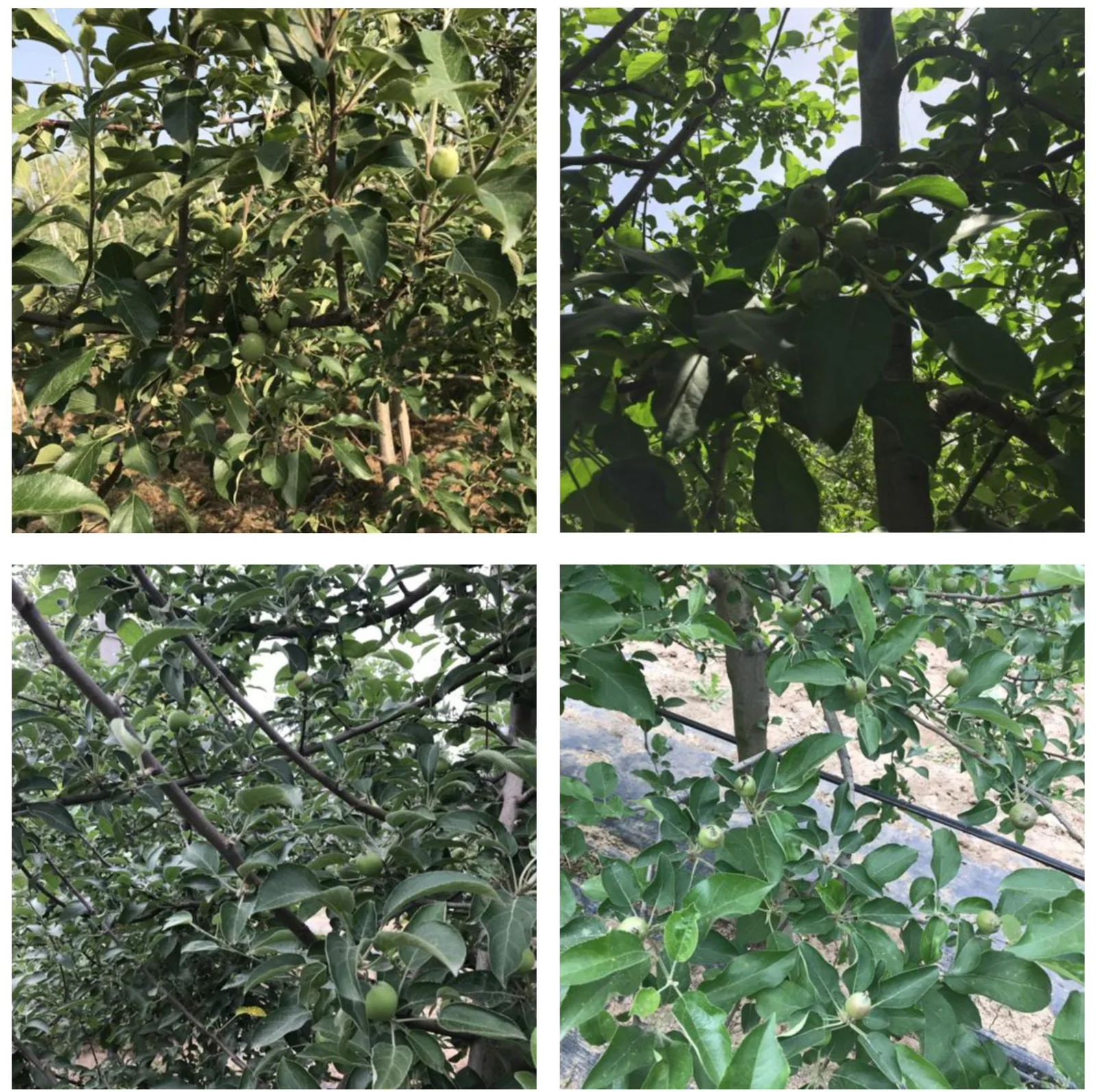
Example images captured in the orchard.

The final dataset comprised 2,517 images captured during the acquisition period (Wang and Wang, 2025). To standardize input dimensions, each image was uniformly rescaled to 500 × 500 pixels. Manual annotation of fruitlets was performed using LabelImg software, with bounding box coordinates archived in XML format. Subsequently, the dataset was randomly partitioned into training (2,013 images), validation (253 images), and test (251 images) subsets at an 8:1:1 ratio. This random splitting strategy was deliberately employed to mitigate selection bias and rigorously evaluate the model’s generalization capability across varied experimental configurations. Detailed statistics regarding the dataset partition are summarized in **Table 1**. To better illustrate the dataset’s characteristics, we visualize the statistical distribution of apple fruitlet instances in **Figure 2**. Following the MS COCO benchmark, the thresholds for small objects (≤32 pixels) and medium objects (32–96 pixels) are annotated on the width/height distributions. Empirically, apple fruitlets in our dataset exhibit a near-square bounding box aspect ratio (≈1:1). This near-1:1 aspect ratio justifies using individual width and height distributions as reliable proxies for overall object scale. As shown in **Figure 2**, the majority of fruitlets exhibit widths and heights below 32 pixels, confirming that the dataset predominantly consists of small objects, a key challenge for detection models. However, a non-negligible number of instances are also observed in the 32–96 pixels range, corresponding to medium-sized fruitlets. This mixed-scale distribution accurately reflects the natural variability in fruit growth stages and imaging perspectives encountered in real-world orchard environments. Such diversity not only enhances the realism of the dataset but also provides a more rigorous testbed for evaluating model robustness across different object scales.

**Table 1:** Summary of the dataset split for apple fruitlet detection.

Subset	Images	Instances
Training	2,013	17,889
Validation	253	2,215
Test	251	2,311
Overall	2,517	22,415

**Figure 2:**
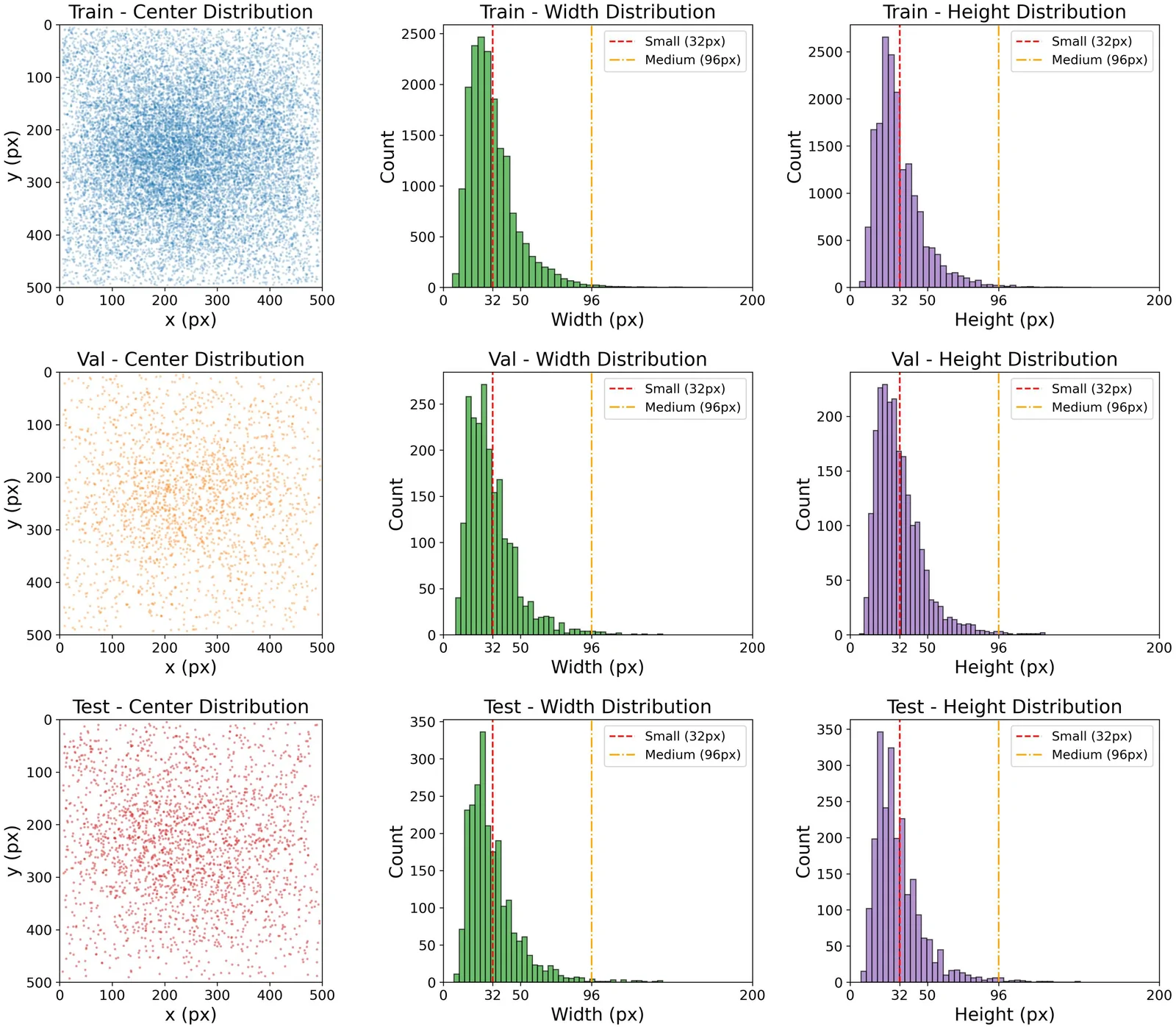
Statistical distribution of apple fruitlet instances in the dataset. (Top row) Training set, (Middle row) Validation set, and (Bottom row) Test set. Left column shows the center coordinates (x, y); middle and right columns show the histograms of width and height, respectively.

The dataset used in this study can be found in https://dx.doi.org/10.21227/z74d-8t41.

### 2.2 Model construction

#### 2.2.1 Overall architecture of the proposed method

In this work, we adopt the RepDown and D3C2f modules from YOLO-ULM as backbone-level building blocks, and the core contribution lies in the task-oriented architectural redesign at the neck level, specifically the asymmetric multi-scale fusion strategy that prioritizes small-object detection while maintaining robust performance across all target scales. This strategy differs fundamentally from a simple combination of existing modules in three aspects. First, the asymmetric allocation of computational resources, enhancing the high-resolution P2 branch while weakening the low-resolution P5 branch, is a task-driven decision based on the statistical analysis of the target scale distribution. Second, the four-scale detection head extends the conventional three-scale design with a dedicated P2 scale, which is essential for detecting extremely small fruitlets that are poorly represented at coarser scales. Third, we demonstrate that the asymmetric neck exhibits a synergistic interaction with the TrueFoCIoU loss, an interaction that is specifically analyzed and exploited in this work. Therefore, this work extends beyond a simple combination of existing modules and presents a novel, task-specific architecture for apple fruitlet detection.

The proposed detector, termed LT-YO-AN, addresses the computational asymmetry inherent in pre-thinning orchards—where small fruitlets are predominant but macro-targets are scarce. Departing from conventional YOLO architectures that allocate resources uniformly across scales, we introduce an asymmetric multi-scale detection framework optimized for small-object scenarios under resource-constrained conditions. As illustrated in **Figure 3**, the architecture comprises three core components: a streamlined backbone, an asymmetric feature pyramid neck, and a four-scale detection head.

**Figure 3:**
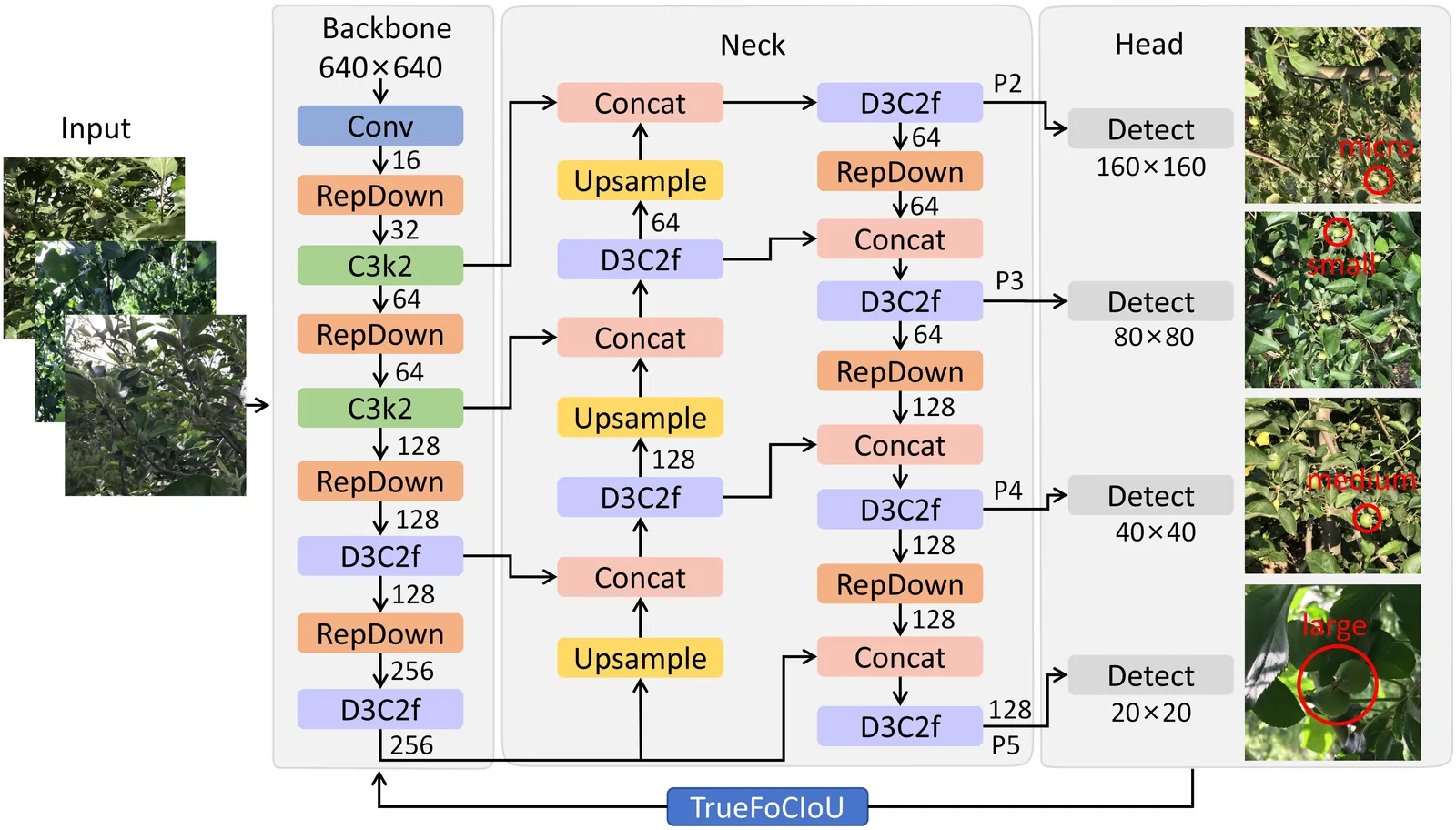
Architecture of the proposed LT-YO-AN model.

To balance representational capacity with efficiency, the backbone utilizes a stack of convolutions and RepDown modules (Conv and RepDown) (**Figure 4**) for hierarchical feature extraction. The lightweight C3k2 and D3C2f modules are adopted as basic building blocks to enhance feature representation while maintaining low latency. Crucially, this design generates four hierarchical feature maps, P2, P3, P4, and P5, corresponding to strides of 4, 8, 16, and 32 (resolutions: 160×160 to 20×20 for 640×640 inputs). By deploying RepDown in deeper stages, we preserve receptive field expansion while mitigating the information loss typically associated with aggressive downsampling.

**Figure 4:**
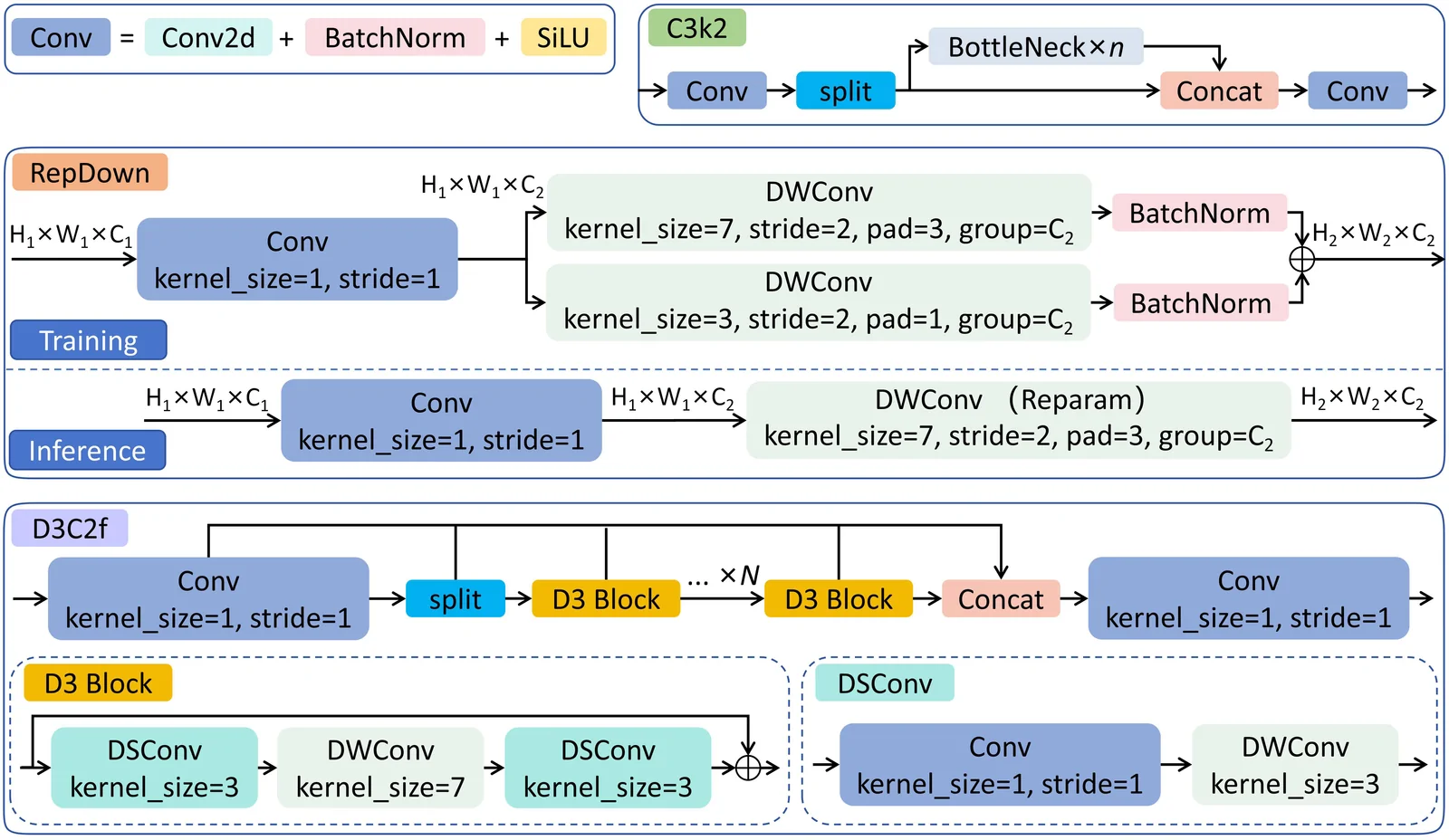
Detailed architectures of the key modules in **Figure 2**.

In the neck, a bidirectional feature pyramid network is constructed to strengthen multi-scale feature fusion. Unlike conventional symmetric designs, we apply an asymmetric channel allocation strategy. On the top-down pathway, deep semantic features from the backbone P5 are progressively upsampled and fused with shallow spatial features through three consecutive Concat–D3C2f stages. Notably, the P2 layer is explicitly enhanced by aggregating high-resolution spatial details with strong semantic cues, thereby improving the discriminability of micro-scale targets. On the bottom-up pathway, the P5 layer is deliberately weakened. The intermediate RepDown layer reduces the channel dimension from 256 to 128, and the final D3C2f block compresses the concatenated feature back to 128 channels before detection. This design significantly alleviates computational redundancy associated with large-object predictions without sacrificing overall performance.

The head adopts a four-scale configuration (P2-P5) to cover a broader spectrum of object scales. Benefiting from the width scaling strategy (width multiplier = 0.25), all convolutional layers, starting with the stem convolution, are uniformly compressed to fit the Nano configuration. Consequently, the effective channel dimensions of the four detection branches are 64, 64, 128, and 128, respectively. This asymmetric yet balanced design ensures that computational resources are preferentially allocated to small- and medium-scale objects, which dominate the target application scenario.

#### 2.2.2 Backbone design

The backbone follows the hierarchical feature extraction paradigm established in recent YOLO-series detectors. Standard convolutional layers (Conv) and C3k2 blocks serve as elementary building units to progressively transform low-level textures into high-level semantics. To meet the stringent latency requirements of edge deployment, we adopt a YOLO-ULM-inspired Nano configuration (Han et al., 2026), employing a width multiplier of 0.25 and a depth multiplier of 0.33. This lean configuration aligns with the limited hardware budget of orchard robots while providing sufficient capacity to model the fine-grained textures of fruitlets. A key distinction of the proposed backbone lies in the integration of RepDown and D3C2f modules, inherited from the ultra-lightweight YOLO-ULM architecture (Han et al., 2026) to further reduce redundancy while preserving representational capacity.

RepDown is implemented as a re-parameterizable downsampling operator that replaces conventional strided convolutions. As illustrated in **Figure 3** (middle), RepDown first applies a pointwise convolution to align the channel dimension. Subsequently, two parallel depthwise convolutional branches with kernel sizes of 7×7 and 3×3 (both stride 2) are executed independently, each followed by batch normalization. Their outputs are summed to produce the intermediate feature map. Crucially, our implementation supports dynamic input channel adaptation during the forward pass. If the incoming channel count mismatches the pretrained pointwise convolution, the convolutional weight is reinitialized on the fly without introducing new modules, thereby avoiding lazy initialization issues. During inference, RepDown switches to a deploy mode in which the 3×3 depthwise kernel is zero-padded to 7×7, and its weights and biases are fused with those of the 7×7 branch. This results in a single equivalent 7×7 depthwise convolution, preserving multi-scale representation while achieving plain-convolution efficiency. Such re-parameterization is particularly advantageous in later backbone stages, where aggressive downsampling typically incurs irreversible information loss critical for small-target detection.

The D3C2f module serves as an efficient alternative to standard C3k2 blocks in deeper stages. Inspired by the D3 block design in YOLO-ULM (Han et al., 2026), each D3 block comprises two 3×3 depthwise separable convolutions (DSConv), a 7×7 depthwise convolution, and a residual connection, thereby enhancing both local detail and large-context modeling. As depicted in **Figure 4** (bottom), D3C2f inherits the split-aggregate topology of the C2f architecture. The input feature map is first processed by a pointwise convolution and split into two parts along the channel dimension. One part is directly reserved for the final concatenation, while the other is sequentially refined by multiple D3 blocks. Crucially, the intermediate channel dimension within each D3 block is compressed to C=e*C_2_ with e=0.5, where C_2_ denotes the output channel dimension of the D3C2f module. All processed features are subsequently concatenated and fused by a final 1×1 convolution. This compressed intra-block representation substantially reduces computational redundancy while preserving high-level semantic abstraction. By constraining intra-block channels, computational resources are reallocated from redundant feature transformations to the preservation of high-resolution spatial details essential for discriminating diminutive fruits.

By stacking RepDown and D3C2f modules in later backbone stages, the proposed architecture generates four robust hierarchical feature maps, P2, P3, P4, and P5, which form the foundation for subsequent asymmetric feature fusion in the neck. Under the adopted Nano configuration, these feature maps possess channel dimensions of 64, 128, 128, and 256, respectively.

#### 2.2.3 Neck and asymmetric feature fusion

To enrich multi-scale representations, we construct a bidirectional feature pyramid network that propagates semantic information top-down and spatial details bottom-up. Unlike conventional symmetric necks, we introduce an asymmetric feature fusion strategy tailored for edge deployment and the predominance of small-object.

On the top-down pathway, deep semantic features from the backbone P5 are progressively upsampled and fused with shallow features via three Concat-D3C2f stages. Notably, the P2 feature map is explicitly reinforced by concatenating it with upsampled high-level semantics and refining the result through an additional D3C2f block, thereby strengthening the discriminability of micro-scale objects.

On the bottom-up pathway, the neck propagates refined shallow features back to deeper layers to complement localization cues. To alleviate computational redundancy associated with large-object detection, we deliberately weaken the P5 branch. Specifically, the intermediate RepDown layer reduces the channel dimension from 1024 to 512 before concatenation with the backbone P5 feature. The subsequent D3C2f block further compresses the fused representation to 512 channels prior to detection. Under the adopted Nano configuration (width multiplier = 0.25), the fused feature maps produced by the neck possess channel dimensions of 64, 64, 128, and 128 for P2, P3, P4, and P5, respectively. This design substantially reduces floating-point operations (FLOPs) without sacrificing the recall of large-scale instances.

The asymmetric neck structure is motivated by the following theoretical considerations. First, from the perspective of information flow, the gradient of the detection loss with respect to the feature maps at different scales is proportional to the number of targets assigned to that scale. Since the majority of apple fruitlets in our dataset are small objects (width/height < 32 pixels, as shown in **Figure 2**), the P2 branch carries the dominant gradient signal for optimizing the detector. By strengthening the P2 branch, the asymmetric neck amplifies this gradient signal, leading to more efficient optimization for small-object detection. Conversely, the P5 branch, which corresponds to large objects that are rare in our dataset, contributes less gradient information; therefore, weakening this branch reduces redundant computation without sacrificing detection accuracy. From the perspective of feature complementarity, the P2 feature map preserves fine-grained spatial details (e.g., edges and texture) that are essential for distinguishing small fruitlets from complex orchard backgrounds (e.g., leaves and branches), while the P5 feature map provides high-level semantic context that is less critical for this task. The asymmetric allocation of channels across scales follows the principle of allocating computational resources where they are most needed, thereby maximizing the signal-to-noise ratio of the feature representation for the target scale distribution. Furthermore, from the perspective of spatial resolution matching, the P2 branch provides a higher-resolution feature map (stride of 4) compared to the P5 branch (stride of 32). For apple fruitlets with widths and heights predominantly below 32 pixels, the P2 branch allocates approximately 8×8 feature pixels per target, preserving sufficient spatial information for accurate localization. In contrast, the P5 branch would represent such targets with fewer than 1 feature pixel, leading to severe information loss. Therefore, enhancing the P2 branch ensures that small targets are represented with adequate spatial resolution, avoiding quantization errors and improving localization accuracy. Finally, from the perspective of complexity-optimality trade-off, the asymmetric design redistributes the computational budget from the less informative P5 branch to the more critical P2 branch, achieving a better accuracy-efficiency balance.

#### 2.2.4 Detection head and loss function

The detector employs a four-scale decoupled head that takes the multi-scale feature maps from the neck, namely P2, P3, P4, and P5, as inputs. Compared with conventional three-scale configurations, the inclusion of the high-resolution P2 branch significantly improves the localization accuracy for micro-scale objects, which are often under-represented in standard benchmarks. Under the Nano configuration, these feature maps possess actual channel dimensions of 64, 64, 128, and 128, respectively, ensuring a balanced computational budget across scales. Each detection head independently predicts bounding boxes, objectness scores, and class probabilities.

For bounding box regression, we employ a Focal-weighted CIoU loss (implemented as TrueFoCIoU) (Zhang et al., 2026). This formulation integrates the geometric constraints of CIoU with a focal re-weighting mechanism, introducing a dynamic term *IoU^γ^* to suppress gradients from well-localized samples and emphasize hard-to-detect instances. The loss is defined as Equation 1.

(1) $$L_{TrueFoCIoU}=IoU^{\gamma }\cdot L_{CIoU}$$

where *γ* is a tunable focusing parameter (set to 0.5 in this work) and *L_CIoU_* denotes the standard Complete IoU loss incorporating center-distance and aspect-ratio penalties.

Objectness and classification are optimized using Binary Cross-Entropy (BCE) loss, denoted as $L_{{BC}_{E}}^{obj}$and $L_{{BC}_{E}}^{cls}$, respectively. The total training loss is formulated as Equation 2.

(2) $$L_{total}=\lambda_{box}L_{TrueFoCIoU}+\lambda_{obj}L_{BCE}^{obj}+\lambda_{cls}L_{BCE}^{cls}$$

where *λ_box_* = 7.5, *λ_obj_* = 1.0, and *λ_cls_* = 0.5 following the default YOLO training protocol. Furthermore, the Distribution Focal Loss (DFL) (Li et al., 2022) is incorporated to model bounding box regression as a discrete distribution, which further stabilizes localization under the Nano configuration.

## 3 Experimental setup and evaluation metrics

### 3.1 Hardware and software configuration

All experiments were conducted on a desktop workstation equipped with an Intel Core i7-14700 processor (20 cores, 28 logical threads, base frequency 2.10 GHz, x86_64 architecture) and 32 GB of system memory, running the Windows 11 operating system. The computational workload was accelerated by an NVIDIA GeForce RTX 5060 GPU with CUDA Version 13.2. The software environment was built on Python 3.12.12, with the deep learning framework PyTorch 2.9.0+cu128 installed to leverage GPU-accelerated tensor operations. The Ultralytics YOLO implementation was adopted as the codebase, and all models were trained and inferred under consistent environmental conditions to ensure reproducibility and fair comparison.

### 3.2 Training configuration

To evaluate the effectiveness of our proposed LT-YO-AN architecture, all models were trained from scratch for 200 epochs to ensure a fair comparison of architectural design. All models were trained under identical hyperparameter settings, including a batch size of 8, an initial learning rate of 0.01, a final learning rate factor of 0.01, a weight decay of 0.0005. A warm-up strategy was applied for the first 3 epochs to stabilize the early training phase. The learning rate decayed following a linear schedule. For the optimizer, we followed each model’s official configuration to ensure all models operate under their recommended training settings. Specifically, the proposed LT-YO-AN and its baseline YOLOv12n were optimized using SGD with a momentum of 0.937, consistent with the YOLOv12 official implementation. The remaining baseline models (YOLOv8n, YOLOv9t, YOLOv10n, YOLOv11n, and YOLOv26) adopted the auto optimizer setting from their respective official releases, which automatically selects the most suitable optimizer for each architecture. This ensures that the comparison reflects genuine architectural differences rather than suboptimal hyperparameter choices.

### 3.3 Evaluation metrics

To comprehensively evaluate detection accuracy and computational efficiency, we adopt a suite of evaluation metrics. Precision (P) and Recall (R) measure the correctness of positive predictions and the coverage of ground-truth instances, respectively, while the F1-score is their harmonic mean, providing a balanced measure between them. The Precision (P), Recall (R) and F1-score can be calculated using Equations 3–5, respectively.

(3) Precision=TP/(TP+FP)

(4) Recall=TP/(TP+FN)

(5) F1=2·Precision·Recall/(Precision+Recall)

where *TP* denotes true positives, *FP* denotes false positives and *FN* denotes false negatives.

The primary detection accuracy metric is mean Average Precision (mAP). Specifically, mAP50 denotes the average precision (AP) calculated at a single Intersection-over-Union (IoU) threshold of 0.5, whereas mAP50–95 is the principal COCO metric, computed by averaging the AP values over ten IoU thresholds ranging from 0.5 to 0.95 with a step size of 0.05. Formally, mAP is defined as Equation 6.

(6) $$mAP=\frac{1}{N}\sum_{i=1}^{N}AP_{i}$$

where *N* is the number of categories.

Computational efficiency is assessed using Inference Latency (milliseconds per image), Model Size (MB), Parameters (×10^6^), GFLOPs, and Network Depth (number of layers). These metrics collectively characterize the trade-off between accuracy and resource consumption, which is critical for edge deployment.

## 4 Results and discussions

### 4.1 Detection performance of our proposed model

#### 4.1.1 Training convergence

**Figure 5** illustrates the training and validation curves for key metrics, including box loss, classification loss, distribution focal loss, precision, recall, mAP50, and mAP50-95. All loss curves exhibit smooth convergence with minimal overfitting, as evidenced by the close alignment between training and validation trends. Specifically, both training and validation box losses (train/box_loss and val/box_loss in **Figure 5**) decrease from approximately 2.25 to 0.9, indicating stable bounding box regression. Similarly, the training and validation classification losses (train/cls_loss and val/cls_loss in **Figure 5**) drop from around 4.0 to 0.7, demonstrating effective class discrimination. The training and validation distribution focal losses (train/dfl_loss and val/dfl_loss in **Figure 5**) stabilize at roughly 1.5, confirming robust localization through distribution-based learning. Meanwhile, precision and recall (metrics/precision(B) and metrics/recall(B) in **Figure 5**) increase rapidly to values above 0.8, while mAP50 and mAP50–95 converge to 0.9 and 0.6 (metrics/mAP50(B) and metrics/-95(B) in **Figure 5**), respectively. These trends reflect consistent performance improvement throughout the training process and confirm the stability of the proposed architecture under the configured hyperparameters.

**Figure 5:**
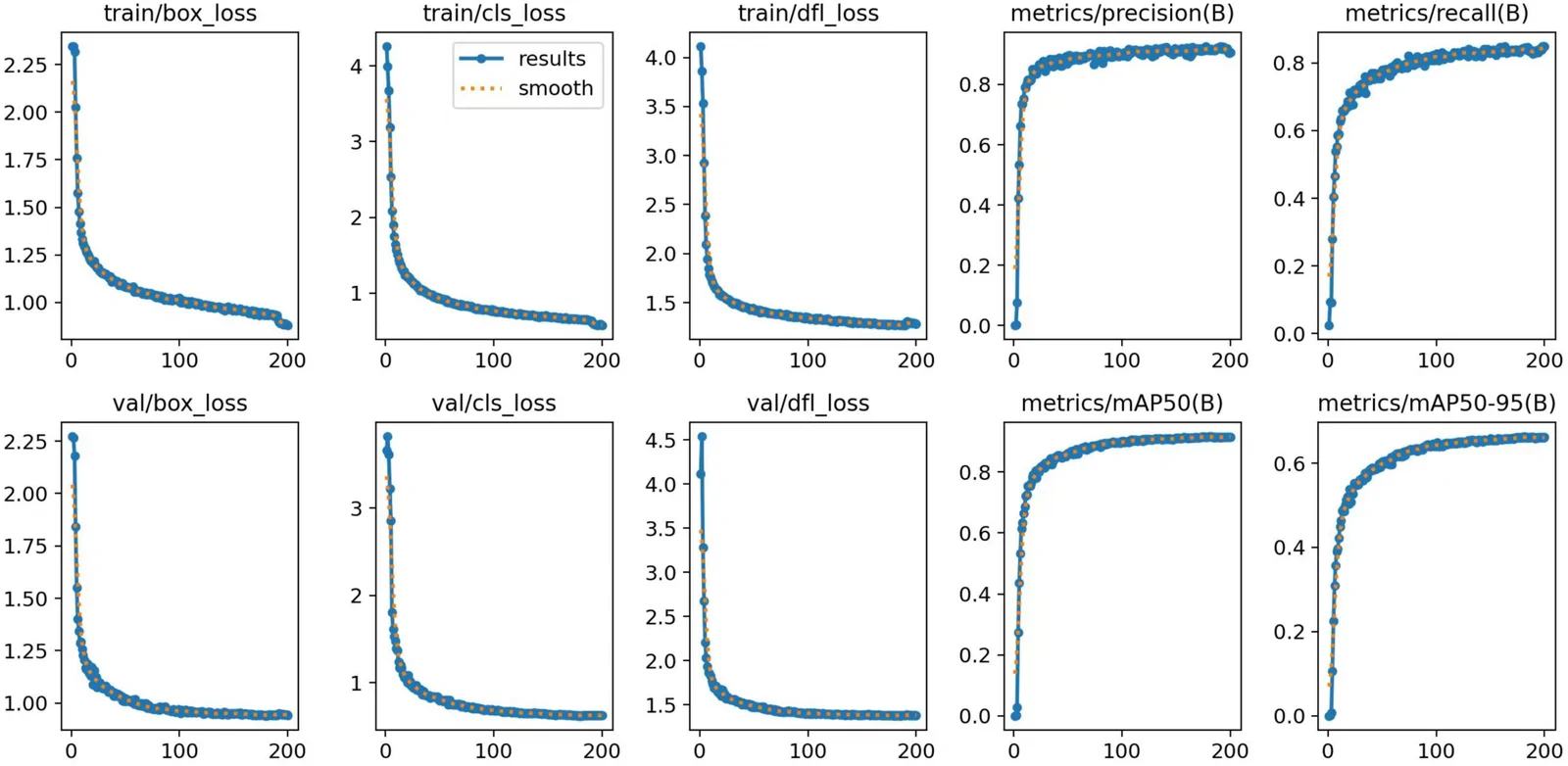
Training and validation curves for box loss (train/box_loss and val/box_loss), classification loss (train/cls_loss and val/cls_loss), distribution focal loss (train/dfl_loss and val/dfl_loss), precision (metrics/precision(B)), recall (metrics/recall(B)), mAP50 (metrics/mAP50(B)), and mAP50-95 (metrics/mAP50-95(B)).

#### 4.1.2 Detection performance

The detection results on the test set are summarized in **Table 2**. Our approach achieves a precision of 0.899, a recall of 0.860, and an F1-score of 0.879, indicating a balanced performance between false positives and false negatives. Regarding object detection benchmarks, the model obtains an mAP50 of 0.928, demonstrating high accuracy for relatively loose localization at an IoU threshold of 0.5. Furthermore, the mAP50–95 reaches 0.675, reflecting strong performance under stricter IoU thresholds up to 0.95 and indicating precise bounding box regression. In terms of model efficiency, the proposed network contains 1.49 million learnable parameters, resulting in a compact model size of 3.5 MB on disk. With a computational complexity of 11.5 GFLOPs, this lightweight profile renders the model highly suitable for deployment on resource-constrained edge devices, such as unmanned aerial vehicles (UAVs) and ground-based agricultural robots, within agricultural scenarios.

**Table 2:** Detection performance of our LT-YO-AN methods.

Networks	Precision	Recall	F1	mAP50	mAP50-95	Model Size/MB	Parameters/×10^6^	GFLOPs
LT-YO-AN	0.899	0.860	0.879	0.928	0.675	3.5	1.49	11.5

**Figure 6** visualizes representative detection results of the proposed model across four distinct illumination conditions: sunny direct sunlight (top-left), sunny backlight(top-right), cloudy direct sunlight(bottom-left), and cloudy backlight scenarios (bottom-right). These examples demonstrate the model’s robustness in handling variations of lighting intensity and directionality commonly encountered in real-world orchard environments. A detailed inspection reveals three distinct strengths. First, the model exhibits reliable performance in handling occlusions, accurately localizing fruits partially obscured by foliage and other fruits even under challenging backlighting conditions where contrast is significantly reduced (bottom-right subfigures in **Figure 6**). This resilience is largely attributed to the asymmetric neck, which enhances multi-scale feature fusion to preserve contextual information despite adverse lighting. Second, the model effectively identifies small-scale targets, such as fruits in cluttered backgrounds, across all illumination settings. This capability is primarily enabled by the integration of the high-resolution P2 layer within the asymmetric neck, which retains fine spatial details essential for discriminating diminutive fruits. The D3C2f module further refines these shallow features through efficient hierarchical representation, complementing the neck’s design to maintain sensitivity to small objects. Third, the predictions marked by red boxes predominantly achieve high confidence scores exceeding 0.7, while exhibiting minimal spurious detections throughout the diverse lighting scenarios. The visual results confirm consistent detection quality from bright sunny days to overcast conditions, and from direct sunlight to backlight orientations. In summary, these findings corroborate the evaluation metrics, confirming that the proposed architecture delivers robust and stable performance across complex illumination conditions in agricultural environments.

**Figure 6:**
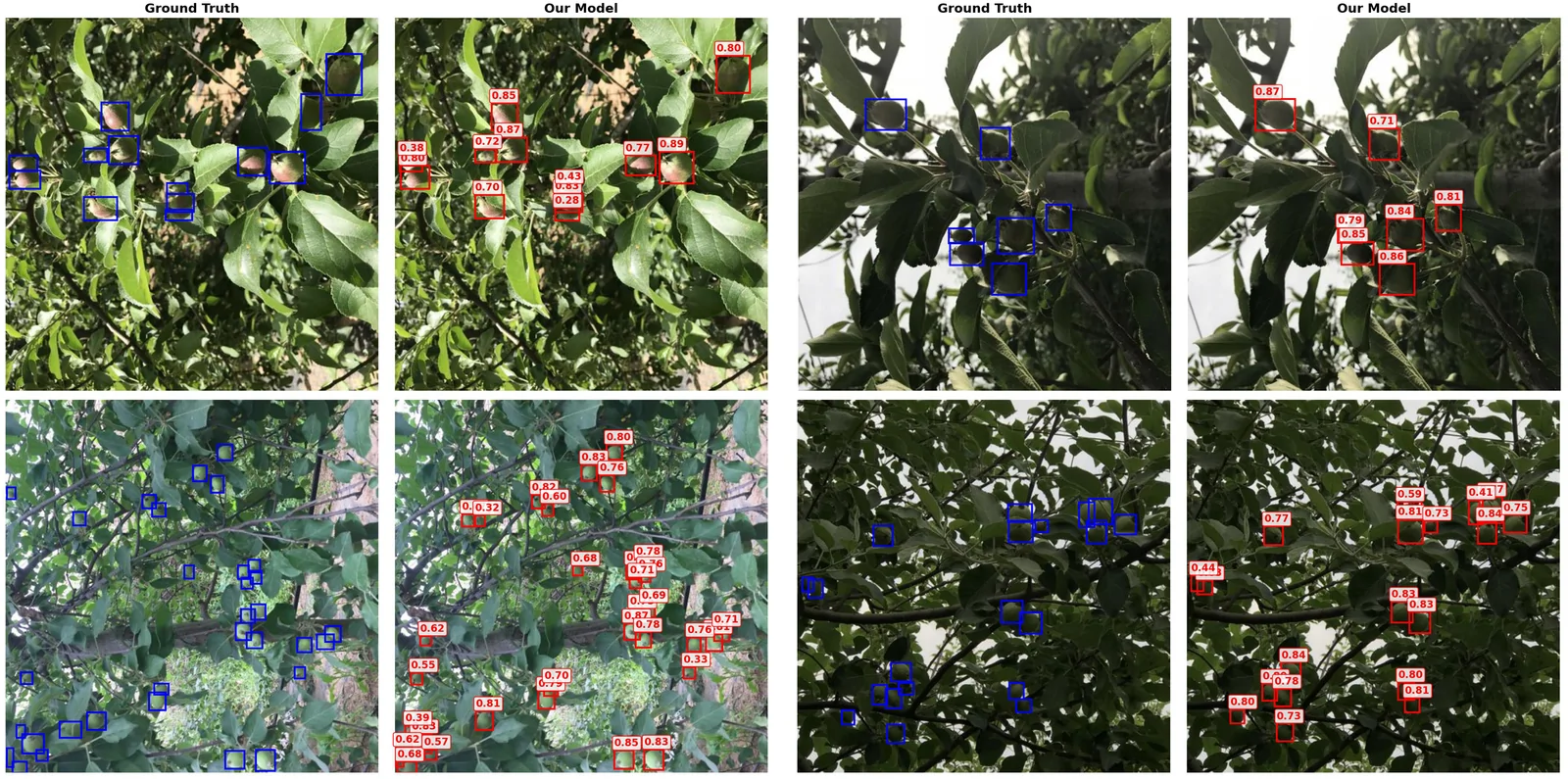
Representative detection results of the proposed model under four illumination conditions: sunny direct sunlight (top-left), sunny backlight (top-right), cloudy direct sunlight (bottom-left), and cloudy backlight (bottom-right). Blue boxes denote ground truth annotations, and red boxes indicate predictions generated by the proposed model.

### 4.2 Comparative evaluation with state-of-the-art methods

To rigorously validate the effectiveness of the proposed LT-YO-AN architecture, comprehensive comparative experiments were conducted against six recent YOLO variants: YOLOv8n (Varghese and Sambath, 2024), YOLOv9t (Wang et al., 2024b), YOLOv10n (Wang et al., 2024a), YOLOv11n (Khanam and Hussain, 2024), YOLOv12n (Tian et al., 2026), and YOLOv26 (Sapkota et al., 2025). These models represent successive generations of lightweight detectors, making them highly relevant baselines for real-time agricultural applications. All models were trained under identical hyperparameter configurations to ensure a fair comparison. Training was performed on a single RTX 5060 GPU with a batch size of 8, a learning rate of 0.01, and 200 epochs. No pre-trained weights were employed to eliminate potential biases from external data.

**Figure 7** illustrates the training dynamics across six key metrics over 200 epochs, including train loss, validation loss, precision, recall, mAP50, and mAP50-95. A magnified view of epochs 133~200 reveals critical convergence behaviors. For train loss, all models exhibit a similar downward trend and eventually stabilize. For validation loss, YOLOv26n achieves the lowest loss, while YOLOv10n remains the highest. Regarding precision, all curves show continuous oscillation throughout training, with YOLOv9t and YOLOv10n consistently positioned at the bottom of the fluctuation range, indicating lower classification stability. In contrast, our model maintains a mid-to-upper range, demonstrating more robust precision performance. For recall, all models’ curves overlap and oscillate together, showing no clear dominance by any single model, though our method remains slightly above the average. Most importantly, in mAP50 and mAP50-95, our model consistently stays at the top of all curves, significantly outperforming all baseline variants. This indicates that, despite similar loss trends and overlapping recall/precision, our architecture delivers superior overall detection quality, particularly in balancing localization and classification accuracy.

**Figure 7:**
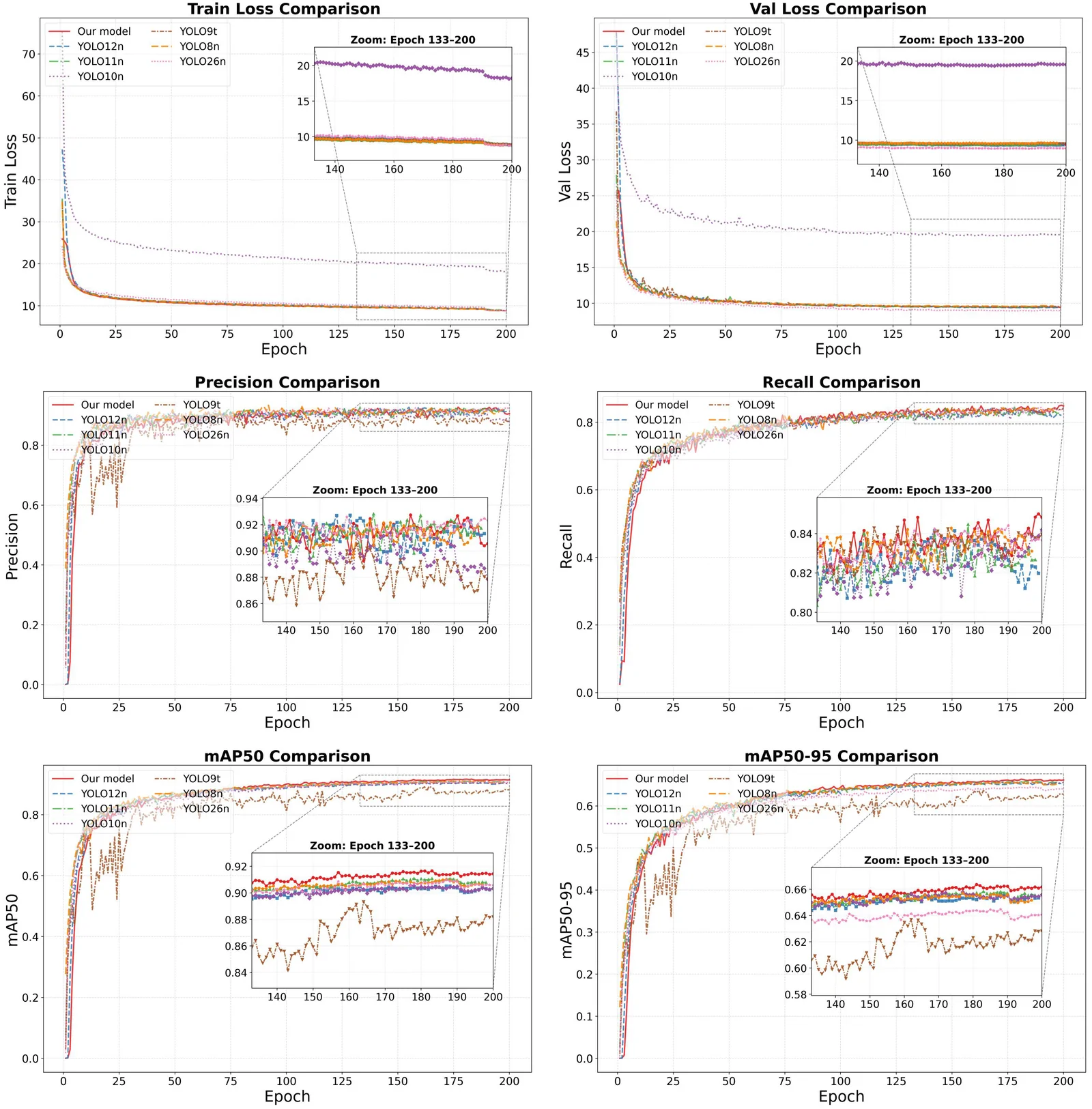
Training and validation performance curves of the proposed model and baseline YOLO variants across 200 epochs.

To further evaluate the generalization capability, all seven models were tested on the test set, and the results are summarized in **Table 3**. The proposed model achieves the highest mAP50 and mAP50–95 scores, while maintaining the smallest model size (3.5 MB) and lowest parameter count (1.49 M). Although LT-YO-AN exhibits the second-lowest precision (0.899) among the comparison methods (only higher than YOLOv9t), this is offset by the highest recall (0.860) and strongest mAP50-95 (0.675), yielding a competitive F1-score of 0.879. While the model incurs increased computational complexity (11.5 GFLOPs), this is justified by substantial gains in detection coverage (highest recall) and localization accuracy (highest mAP50-95), rendering it well-suited for orchard deployment scenarios.

**Table 3:** Performance of seven methods.

Networks	Precision	Recall	F1	mAP50	mAP50-95	Model Size/MB	Parameters/×10^6^	GFLOPs
YOLOv8n	0.921	0.840	0.879	0.923	0.662	6.0	3.00	8.1
YOLOv9t	0.895	0.848	0.871	0.887	0.624	4.4	1.97	7.6
YOLOv10n	0.904	0.857	0.880	0.923	0.665	5.5	2.27	6.6
YOLOv11n	**0.928**	0.836	0.880	0.924	0.663	5.2	2.58	6.3
YOLOv12n	0.923	0.837	0.878	0.920	0.660	6.0	2.56	6.3
YOLOv26	0.918	0.854	**0.885**	0.925	0.655	5.2	2.37	**5.3**
LT-YO-AN (Ours)	0.899	**0.860**	0.879	**0.928**	**0.675**	**3.5**	**1.49**	11.5

Bold font indicates the highest or lowest value in each column.

Visual comparisons under diverse illumination conditions in **Figure 8** further support these findings. In the first row of **Figure 8**, the proposed model successfully detects extremely small fruits that are entirely missed by the other methods. In the second row of **Figure 8**, it identifies fruits with weak visual features, whereas YOLOv12n and YOLOv9t fail to detect them, and YOLOv8n, YOLOv11n, YOLOv26 and YOLOv10n produce low-confidence predictions. In the third and fourth rows of **Figure 8**, the proposed model generates noticeably fewer false positives compared with the baseline models, which frequently misclassify tree branches and leaves with similar appearances as fruits. Overall, these visual results explain the advantage in mAP50-95, which penalizes both missed detections and localization errors, and confirm that the proposed architecture is particularly robust to small targets and background clutter.

**Figure 8:**
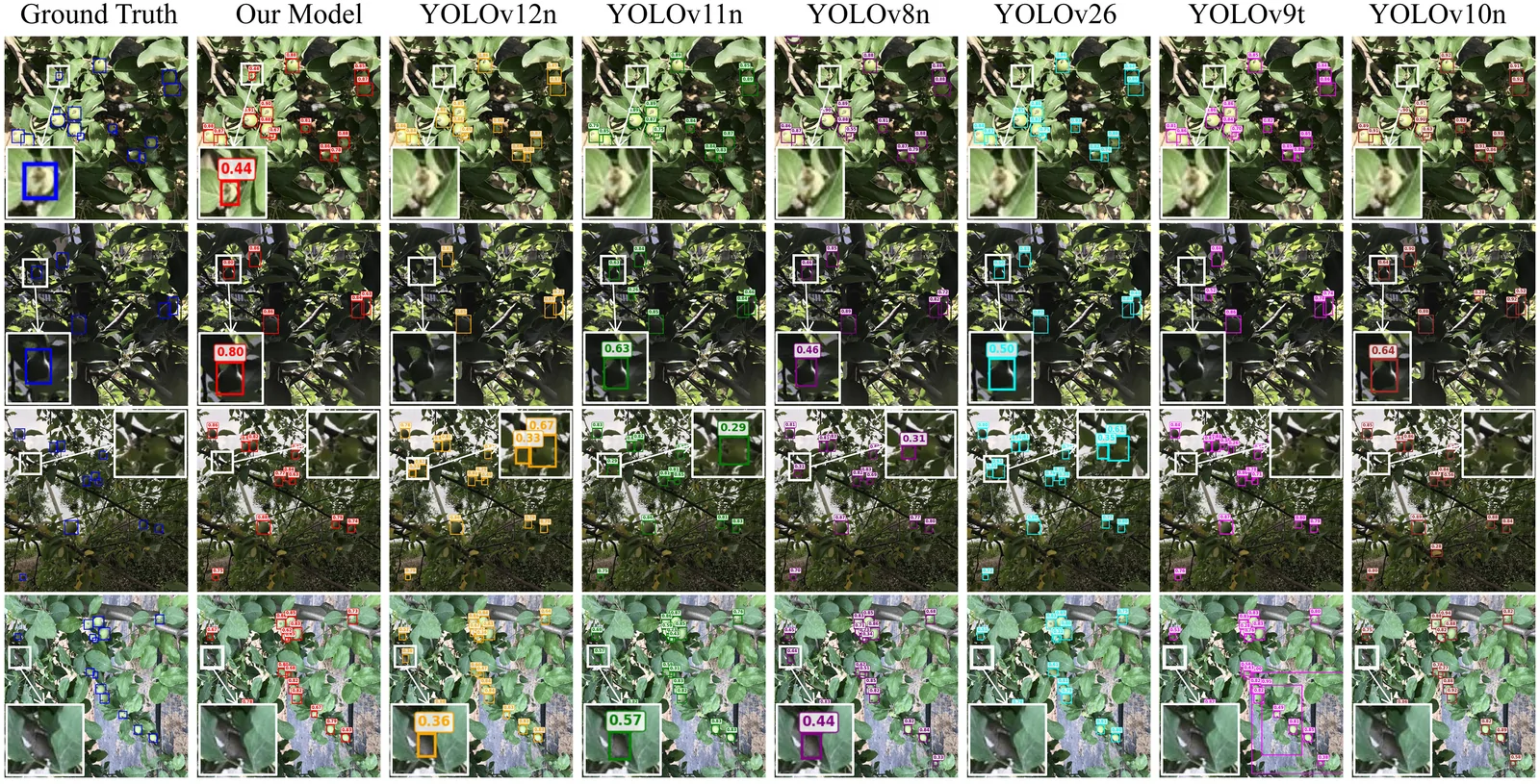
Representative detection results under different illumination conditions.

In summary, the proposed LT-YO-AN achieves the best overall performance and robustness across diverse lighting conditions, characterized by the highest recall and mAP scores despite a marginal drop in precision and increased computational cost. This trade-off positions it as a compelling solution for real-world orchard monitoring, where maximizing fruit detection coverage under environmental variability is paramount.

### 4.3 Ablation experiments

To evaluate the contribution of each proposed component, an ablation study was conducted based on the YOLOv12n baseline, and the results are summarized in **Table 4**. The baseline model (YOLOv12n) achieves a mAP50–95 of 0.660 with 6.0 MB model size and an inference speed of 3.2 ms per image.

**Table 4:** Ablation experiments.

Evaluation metrics	YOLOv12n	LT-YO	LT-AN	LT-TF	LT-YO-AN (Ours)
RepDown + D3C2f		✓	✓	✓	✓
Asymmetric Neck			✓		✓
TrueFoCIoU				✓	✓
Precision	**0.923**	0.904	0.915	0.910	0.899
Recall	0.837	0.836	0.839	0.844	**0.860**
F1	0.878	0.869	0.875	0.876	**0.879**
mAP50	0.920	0.918	0.920	0.921	**0.928**
mAP50-95	0.660	0.662	0.670	0.660	**0.675**
Inference ms/image	3.2	3.3	6.7	**3.0**	6.4
Model Size/MB	6.0	**3.4**	3.5	3.6	3.5
Parameters/×10^6^	2.56	1.57	**1.49**	1.57	**1.49**
GFLOPs	6.3	**5.3**	11.5	**5.3**	11.5
Layers	**159**	163	198	163	198

Bold font indicates the highest or lowest value in each row.

The introduction of RepDown and D3C2f modules (LT-YO, Column 2) significantly reduces the model size from 6.0 MB to 3.4 MB and the parameter count from 2.56 M to 1.57 M, while maintaining nearly identical detection accuracy (an mAP50–95 of 0.662). This demonstrates the effectiveness of these modules in compressing the network without compromising representational capacity. When the asymmetric neck is further integrated (LT-AN, Column 3), the model size remains compact at 3.5 MB, but the inference latency increases to 6.7 ms due to the added computational overhead of high-resolution feature processing. However, this trade-off yields a higher recall (0.839) and mAP50-95 (0.670), indicating that the enhanced P2 feature fusion substantially improves small target sensitivity. In Column 4 (LT-TF), where TrueFoCIoU is applied, the performance slightly fluctuates compared to the baseline, with a marginal decrease in F1-score (0.876), suggesting that the loss function alone provides limited gains without structural enhancements.

The proposed model (LT-YO-AN, Column 5) integrates all three components, achieving the best overall balance between accuracy and efficiency for orchard deployment. Notably, it reaches the highest mAP50 (0.928) and mAP50-95 (0.675) across all configurations, alongside a significant improvement in recall (0.860). Although the inference latency (6.4 ms) and GFLOPs (11.5) are higher than the baseline, the model maintains a minimal size of 3.5 MB and 1.49 M parameters. This indicates that while the asymmetric neck introduce additional computation, the substantial gains in localization accuracy justify the computational expense. Overall, the ablation results confirm that the synergy of structural lightweight design (RepDown+D3C2f), multi-scale feature optimization (asymmetric neck), and refined regression loss (TrueFoCIoU) is crucial for achieving robust performance in complex agricultural scenarios.

## 5 Discussions

### 5.1 Detection performance analysis

The ablation study (**Table 4**) clarifies the distinct role of each component. The integration of RepDown and D3C2f modules primarily drives model compression, reducing the parameter count from 2.56 M to 1.49 M and the model size to a mere 3.5 MB. This substantial reduction is achieved without significant performance degradation, as the re-parameterized structure preserves representational capacity. However, the most significant improvement in localization accuracy, particularly for small fruits, stems from the asymmetric neck. By enhancing the P2 feature map and strategically weakening the P5 branch, the model reallocates computational resources to high-resolution features critical for small target detection. This explains the notable increase in recall (0.860) and mAP50-95 (0.675) in the full model. Conversely, the TrueFoCIoU loss function, while beneficial for refining bounding box regression, contributes less dramatically when applied in isolation. Its primary value emerges in synergy with the structural enhancements, where improved feature extraction provides a richer foundation for the loss function to optimize.

As shown in **Table 4**, the proposed LT-YO-AN achieves the highest recall (0.860) among all comparative models, while exhibiting the second lowest precision (0.899). This high-recall, low-precision pattern indicates that the proposed method successfully detects more true fruitlets, including those that are heavily occluded or exhibit low contrast against the complex orchard background, at the cost of introducing a higher number of false positives. The implications of this trade-off depend on the specific orchard application. For fruit yield estimation, a high recall is generally more desirable, as missing fruitlets (false negatives) can lead to underestimation of the crop load and suboptimal harvesting decisions.

### 5.2 Computational cost analysis

To provide an in-depth understanding of the computational overhead introduced by the proposed asymmetric neck, we analyze the GFLOPs and inference latency of each component based on the ablation experiments in **Table 4**.

As shown in **Table 4**, applying RepDown+D3C2f module (LT-YO vs. YOLOv12n) reduces the GFLOPs from 6.3 to 5.3, demonstrating that this module is computationally efficient. The introduction of the asymmetric neck (LT-AN vs. LT-YO) increases the GFLOPs from 5.3 to 11.5, an increase of 6.2 GFLOPs. This is the primary and sole source of the computational overhead. The increase stems from the newly added P2 branch, which operates on the highest-resolution feature map. The additional convolutional layers and upsampling operations in this branch incur a high computational cost due to the quadratic scaling of convolution operations with spatial resolution. The TrueFoCIoU loss (LT-TF vs. LT-YO) does not affect the computational cost, as it only modifies the loss function.

The inference latency follows a similar trend. The asymmetric neck increases the latency from 3.3 ms to 6.2 ms, while the other components have a negligible impact. This confirms that the computational overhead is concentrated in the high-resolution P2 feature processing.

Although the asymmetric neck increases the computational cost by 6.2 GFLOPs (from 5.3 to 11.5) and the latency by approximately 3.4 ms, it also brings the most significant accuracy improvement. The full model (LT-YO-AN) further improves mAP50 to 0.928 and mAP50–95 to 0.675, achieving the best overall performance. For the target application of apple fruitlet detection, where small-object accuracy is critical, this trade-off is justified. First, the 6.4 ms inference time still meets real-time requirements for most robotic harvesting and monitoring systems. Second, the substantial gains in mAP50-95 (from 0.660 to 0.675) and recall (from 0.837 to 0.860) directly translate to fewer missed fruits, which is of great significance for early yield forecasting and the automation of mechanical fruit thinning. The proposed method delivers the highest accuracy among all lightweight models while maintaining a compact model size of 3.5 MB and 1.49 M parameters, which is suitable for real-time deployment on edge devices.

### 5.3 Synergy analysis of TrueFoCIoU and asymmetric neck

The ablation study in **Table 4** reveals an interesting phenomenon, applying TrueFoCIoU alone (LT-TF) yields minimal performance gains (mAP50-95: 0.660 vs. 0.662 for LT-YO), while its combination with the asymmetric neck (LT-YO-AN) leads to a substantial improvement (mAP50-95: 0.675).

The limited improvement of TrueFoCIoU alone can be attributed to two factors. First, the baseline CIoU loss already provides reasonable gradient signals for bounding box regression. The advantage of TrueFoCIoU lies in its more precise handling of extreme aspect ratios and small object scales. However, when the feature representation is insufficient, the network cannot fully exploit these finer regression signals. The bottleneck lies in the feature quality, not the loss function. Second, small objects are underrepresented in the regression loss. In the baseline architecture, small fruitlets are primarily detected at the P3 and P4 scales, where the feature maps have limited spatial resolution. The regression loss for these objects is computed over a small number of positive samples, and the gradient contribution from these samples is diluted by the dominant large-object samples. Therefore, even with a more precise loss function, the improvement is marginal because the underlying feature representation for small objects is suboptimal.

When TrueFoCIoU is combined with the asymmetric neck, the mAP50–95 improves by 1.3% over LT-YO. This synergy can be explained from two perspectives. First, from a feature-quality complementarity perspective, the asymmetric neck enhances the P2 branch, which provides high-resolution feature maps for small-object detection. This ensures that small fruitlets are represented with sufficient spatial detail, allowing TrueFoCIoU to compute more accurate regression targets. Second, from a gradient allocation complementarity perspective, the asymmetric neck amplifies the gradient signal from small-object supervision by strengthening the P2 branch. When combined with TrueFoCIoU, which provides more precise gradient directions for bounding box regression, the network receives both stronger and more accurate gradients for small-object optimization.

### 5.4 Failure detection case analysis

Despite the robust performance demonstrated in complex orchard environments, the proposed model still exhibits specific failure cases, primarily manifesting as missed detections and false positives. A detailed analysis of these cases, as illustrated in **Figure 9**, provides critical insights into the current limitations.

**Figure 9:**
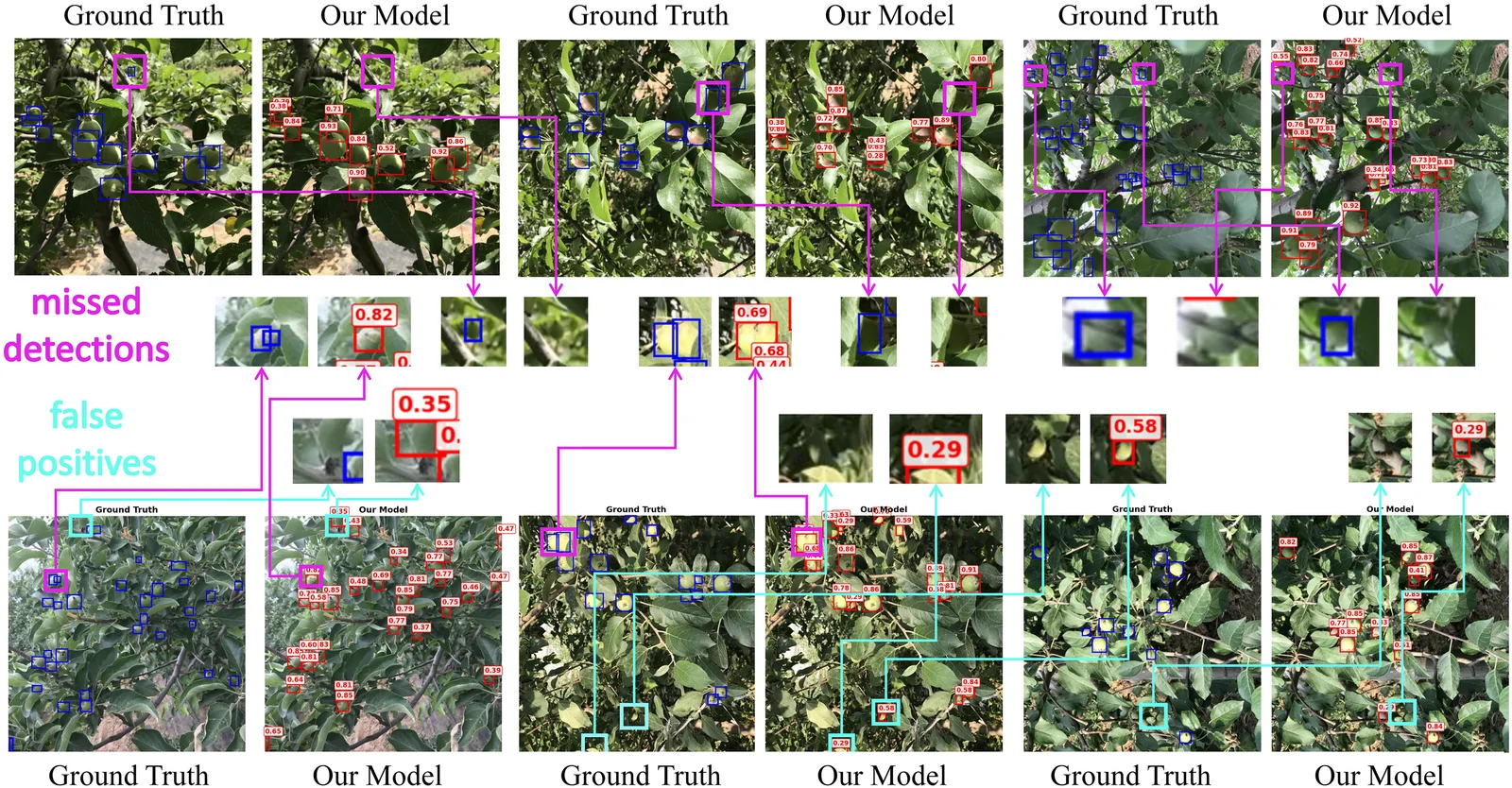
Representative failure cases of the proposed model. Purple and cyan boxes correspond to missed detections and false positives, respectively.

Missed detections predominantly occur under extreme perceptual challenges. The most notable instances involve extremely small, blurred, or severely occluded fruits under low-light conditions. In these scenarios, the target objects lack sufficient discriminative visual features, resulting in a weak response from the network’s feature extraction layers. Notably, many of these challenging samples pose significant difficulties even for human observers, indicating an inherent difficulty in the data distribution rather than solely a model deficiency. Additionally, when multiple fruits overlap with an extremely high degree of intersection, the model occasionally fails to distinguish them as separate instances, erroneously predicting a single bounding box for the entire overlapped region.

False positives, conversely, typically stem from the visual ambiguity between fruits and background elements. The most common source of error involves leaves or branches whose color, texture, or shape closely resemble that of small immature apples. Given the high similarity in low-level features, particularly in dense foliage where small fruits are often partially hidden, the model occasionally misclassifies these background structures as targets.

These failure cases highlight the intrinsic challenges of fruit detection in unstructured natural environments. They underscore that while architectural innovations significantly improve performance, fundamental limitations persist when visual evidence is inherently ambiguous or scarce. Future works will therefore explore the integration of attention mechanisms to better focus on subtle fruit features amidst complex backgrounds. Additionally, self-supervised learning strategies using large amounts of unlabeled orchard data could be employed to improve the model’s robustness to rare and ambiguous cases not well represented in the current training set.

## 6 Conclusions

This study presented LT-YO-AN, a lightweight YOLOv12 framework integrating a RepDown-D3C2f backbone, an asymmetric neck, and the TrueFoCIoU loss function, specifically designed for apple fruitlet detection in structurally complex orchard environments. Comprehensive experiments demonstrated that the proposed model achieved a superior balance between accuracy and efficiency, attaining an mAP50–95 of 0.675 with a compact model size of merely 3.5 MB and 1.49 M parameters. Although the computational complexity (11.5 GFLOPs) was higher than the baseline, the model yielded the highest recall (0.860), mAP50 (0.928), and mAP50-95 (0.675), significantly outperforming benchmark YOLO variants. Failure case analysis highlighted persistent challenges with minute and low-contrast targets, motivating future efforts to enhance feature discrimination and leverage large-scale unlabeled data for improved robustness. Overall, LT-YO-AN provides a computationally efficient and accurate solution for early-stage fruit detection, laying a robust technological foundation for automated fruit thinning and precision orchard management.

## Data Availability

The datasets for this study can be found in the https://dx.doi.org/10.21227/z74d-8t41.
